# 

**Published:** 1888-03

**Authors:** 


### MARCH, 1888.

### CONTENTS.

Original Papers:— *Page*

Droitwich and its Brine Baths By Alfred Crespi, M.R.C.S. ... ... 1

The Treatment of Incomplete Abortion. By A. E. Aust Lawrence, M.D. ... ... ... ... ... ... ... ... ... ... ... ... ... ... 8

Significance of Hoarseness and Aphonia in Cases of Pulmonary Phthisis. By Barclay J. Baron, M.B. Edin. ... ... ... ... ... ... ... 12

Menorrhagia, etc., and Hygienics. By John Meredith, M.D. Edin. ... 17

Clinical Records:—

A Case of Gouty Peripheral Neuritis. By F. W. Jollye, M.R.C.S., L.R.C.P. Lond. ... ... ... ... ... ... ... ... ... ... ... ... 28

Notes from the Out-Patient Department. By W. J. Penny, F.R.C.S.... 36

Reviews of Books ... ... ... ... ... ... ... ... ... ... ... ... ... ... 51

Notes on Preparations for the Sick ... ... ... ... ... ... ... ... 57

Periscope ... ... ... ... ... ... ... ... ... ... ... ... ... ... ... 63

Scraps ... ... ... ... ... ... ... ... ... ... ... ... ... ... ... 71

